# *Stenotrophomonas muris*—first discovered as a potential human pathogen with strong virulence and antibiotic resistance, associated with bloodstream infections

**DOI:** 10.1128/spectrum.02770-24

**Published:** 2025-09-23

**Authors:** Jiaying Liu, Xu Dong, Yanghui Xiang, Yi Li, Yuyun Yu, Tiantian Wu, Xin Yuan, Dan Cao, Hanyin Zhang, Lixia Zhu, Ying Zhang

**Affiliations:** 1 State Key Laboratory for Diagnosis and Treatment of Infectious Diseases, The First Affiliated Hospital, Zhejiang University School of Medicine, Hangzhou, China; 2 Department of Rheumatology, The First Affiliated Hospital, Zhejiang University School of Medicine, Hangzhou, China; 3 Bone Marrow Transplantation Center, The First Affiliated Hospital, Zhejiang University School of Medicine, Hangzhou, China; 4 Jinan Microecological Biomedicine Shandong Laboratory 661980 , Jinan, Shandong, China; Emory University School of Medicine, Atlanta, Georgia, USA

**Keywords:** *Stenotrophomonas muris*, virulence, virulence genes, *Stenotrophomonas maltophilia*, antibiotic resistance, human pathogens

## Abstract

For the first time, *Stenotrophomonas muris*, whose pathogenic potential for humans has never been reported previously, has been identified to be associated with human infections. In this work, the phenotype of *S. muris* virulence, the potential genes that encode higher virulence of *S. muris*, and host responses to *S. muris* infection were investigated for the first time. It was found that S9 (*S. muris* no. 9, isolated from the patient’s bloodstream infection) was more virulent than both S8 (*S. muris* no. 8, isolated from the patient’s sputum) and S1 (*Stenotrophomonas maltophilia* type strain ATCC13637). Candidate genes that may encode higher virulence of S9 were identified, including *virB6*, *dcm*, *hlyD*, and 14 other genes involved in porphyrin metabolism, pyrimidine metabolism, DNA methylation, two-component system, and biofilm formation. Transcriptome analysis of infected host cells (THP-1 cells) showed that 13 candidate genes involved in host response to hypoxia (*HILPDA*, *FOXH1*, *ANGPTL4*, *SLC2A1*, *HK2*, *ADM*, *CXCR4*, *BNIP3*, *PLAT*, *PLOD2*, *STC2*, *STC1*, and *AK4*) were preferentially upregulated by the more virulent strain S9 over the less virulent S8. Two downregulated genes (*SLC26A11* and *SLC8A1*) involved in monoatomic ion transport and the calcium signaling pathway may also need special attention, as they may be involved in pathogenesis. Antibiotic susceptibility testing indicated that strains S8 and S9 demonstrated high resistance to colistin and polymyxin B, with MIC values surpassing clinical breakpoints. For ceftazidime (a cephalosporin) and levofloxacin (a fluoroquinolone), MIC values were elevated in S8/S9 compared to S1 but remained within the susceptible range (ceftazidime: S8 and S9; levofloxacin: S8) or intermediate category (levofloxacin: S9). Because of the above differences in virulence properties and antibiotic susceptibility, it is critical that *S. muris* be distinguished from *S. maltophilia* in a clinical setting for improved care. This work provides the basis for future studies on pathogenic mechanisms of *S. muris* and for developing improved treatment in the future.

**IMPORTANCE:**

*Stenotrophomonas muris* was first discovered as a potential human pathogen. Since it shares 99.72% similarity of the 16S rRNA to *Stenotrophomonas maltophilia*, conventional diagnostic methods usually classify it as *S. maltophilia* clinically. However, the two human pathogens should be distinguished. The reason is that they have different virulence and different drug susceptibilities, which result in different administrations of drugs to treat their infections. To better distinguish these two pathogens and treat infections of *S. muris*, we investigated the virulence genes, the host response of *S. muris* infections, and susceptibility of *S. muris* to different drugs. The results we show in this paper will guide researchers to identify virulence genes, diagnostic biomarkers, and targets to develop treatment strategies for *S. muris* infections.

## INTRODUCTION

Bacterial virulence refers to the relative ability of a bacterium to cause damage to its host. It is a crucial characteristic that determines the pathogenicity of bacteria, reflecting how effectively a bacterium can infect a host, multiply within the host, and cause disease. It is closely associated with virulence factors. Virulence factors help bacteria enter the host and form a beneficial niche for the invading bacteria, modulate the host defense system to weaken the immune response, help bacteria to multiply in the host and transmit to other hosts, and cause damage to the host. Even if a bacterium has weak virulence, it may be strongly harmful to humans under certain conditions, as in opportunistic pathogens. Opportunistic pathogens are normal bacteria in the host that can usually maintain a good survival balance but cause infections when the balance is broken. Many factors can break the balance, such as decreased immunity and illnesses of the host and change in the colonization site of the bacteria. An example of an opportunistic pathogen is *Stenotrophomonas maltophilia* (1), which is of weak virulence but may cause a high mortality rate. Therefore, when studying a newly discovered pathogen, characterizing its virulence should be one of the primary research objectives.

Unfortunately, bacterial virulence is usually complex because of its high context dependence. The pathogen types and other factors, such as the pH in the host, may have impacts on bacterial virulence. For example, in pulmonary infections, *Pseudomonas aeruginosa* and COVID-19 may enhance the virulence of *S. maltophilia* and show cooperative pathogenicity (2). Biofilm formation can affect bacterial virulence. It has been reported that synergistic multispecies biofilms were formed when several opportunistic pathogens were growing in the same environment (3). The complexity of bacterial virulence brings significant challenges for study. Nevertheless, bacterial virulence is usually encoded in genes, so the genetic and genomic tools can provide great aid in the study of bacterial virulence. With the help of bioinformatics and mutant library screens, and comparative genome sequencing of bacterial pathogens and their non-pathogenic counterparts, we could help identify the virulence factors of a pathogenic bacterium (4).

In this study, we discovered that two clinical isolates named S8 (isolated from a patient’s sputum) and S9 (isolated from the bloodstream infection of another patient), which were initially identified as *S. maltophilia* by standard lab microbial identification by mass spectrometry, had stronger virulence than the *S. maltophilia* type strain ATCC13637 (named S1). We subsequently found that the two more virulent isolates are actually *Stenotrophomonas muris* (5) rather than *S. maltophilia* by whole-genome sequencing (WGS) analysis. We use the tentative nomenclature of *S. muris* for the two strains S8 and S9 that are genetically different from *S. maltophilia*, but the official designation of this species has yet to be formalized. Here, we report the virulence properties and whole-genome sequence of the *S. muris* strains in comparison with *S. maltophilia* as well as unique features of host response to *S. muris* infection.

## RESULTS AND DISCUSSION

### Clinical manifestations of *S. muris* infections

S8 was isolated from the sputum of a patient with confirmed acute lymphoblastic leukemia for 7 months. Admission diagnoses found acute lymphoblastic leukemia, L2 type (with myeloid expression, PH+, and high-risk group), and multiple hepatic cysts, but the breath sounds of both lungs were clear without dry or wet rales. The patient was treated with chemotherapy but developed myelosuppression and lung infection with *S. maltophilia* during the chemotherapy treatment. The patient was then treated with co-trimoxazole and levofloxacin and discharged.

S9 was isolated from the bloodstream infection of a different patient. The admission reason of the patient was skin ulceration on the left upper limb for more than 2 months, amputation for half a month, and fever for 6 days. The patient had a 1-year history of diabetes. The infection was not taken seriously after his injury and developed to the point of amputation, which was completed in another hospital. However, 6 days before being admitted to this hospital, the patient developed a fever, with the highest body temperature reaching 40°C, accompanied by chills and rigors and significant pain at the amputation site of the right upper limb. Pus and exudate reappeared at the incision site of the right upper arm. Subsequently, through metagenomic testing, the pus lesions and the blood samples showed Mucororyza infection and cytomegalovirus infection. Computed tomography of the chest indicated that the infectious focus had invaded the thoracic cavity. A combination of amphotericin B, meropenem, and vancomycin was used for anti-infection treatment. However, in the later stage, septic shock and multiple organ failure still occurred. By analyzing blood samples from the patient, *S. maltophilia* was found and isolated.

### Mass spectrometry and biochemical testing misidentified the two *S. muris* clinical isolates as *S. maltophilia*

In routine clinical microbiology identification of clinical isolates, matrix-assisted laser desorption/ionization-time of flight mass spectrometry (MALDI-TOF MS) identified strains S8 and S9 as *S. maltophilia*. Then, biochemical tests using the bioMerieux API 20E kit also identified S8 and S9 as *S. maltophilia*. According to the database, the probability that S8 and S9 are *S. maltophilia* was 99.3%.

### Cytotoxicity testing by SYTOX Green staining

**Fig 1:**
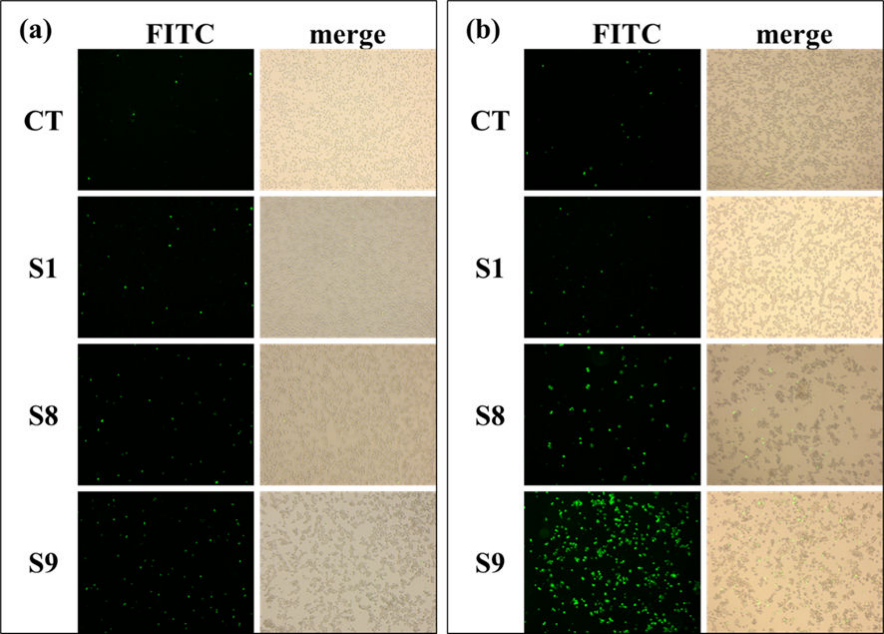
SYTOX Green staining of THP-1-infected cells. THP-1 cells were infected by S1, S8, or S9 bacteria for (**a**) 8 h and (**b**) 18 h and observed under a 10× microscope. “FITC” was shot in fluorescence mode, and “merge” is a merged picture of “FITC” and those shot in normal mode. From the “merge” pictures, the proportion of dead cells could be read with the help of the naked eye. S1, S8, and S9 in the figure are THP-1 cells infected by S1, S8, and S9, respectively. CT is control THP-1 cells without infection.

To determine the cytotoxicity of the isolated strains, the human monocytic leukemia (THP-1) cells were infected with S8, S9, and S1 for 8 h (Fig. 1a) and 18 h (Fig. 1b), respectively, followed by staining with SYTOX Green, a nucleic acid dye that stains cells with compromised plasma membranes but not live cells with intact membranes, making it a useful indicator of dead cells. In both Fig. 1a and b, the control group (CT) had the lowest staining level as expected. The rank of the staining levels for both Fig. 1a and b was S9 > S8 > S1 > CT, which implies that the rank of cytotoxicity or virulence is S9 > S8 > S1. The comparison between Fig. 1a and b also implies that longer bacterial infections resulted in higher mortality of the THP-1 cells, especially for cells infected with S9.

### Lactate dehydrogenase release assay

**Fig 2:**
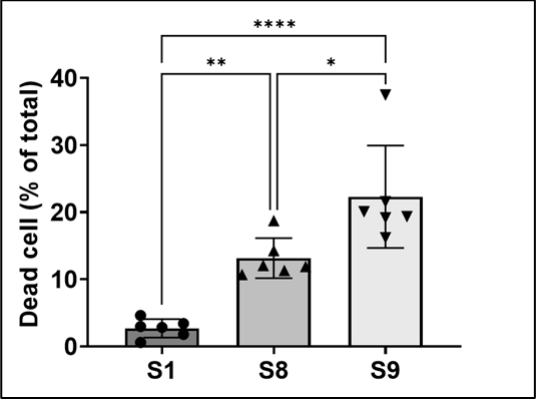
LDH release of infected THP-1 cells. LDH release of THP-1 cells after 24 h infection with S1, S8, or S9 at a multiplicity of infection = 1:1. The death rates of the infected cells were calculated according to the simplified formula *M*_cell_ (%) = (*A*_*e*_ − *A*_*c*_)/(*A*_*m*_ − *A*_*c*_) × 100%, where A_c_ is an averaged value. In this figure, analysis of variance (ANOVA) was used for statistical analysis. The death rate of S1, S8, and S9 is 1.25%–4.15%, 10.03%–16.30%, and 14.30%–30.32% (95% CI), respectively. The death rates of S1 and S9 had significant differences (*P* < 0.0001).

Lactate dehydrogenase (LDH) release assay as a surrogate marker for cell viability can be used to reflect the degree of cell death upon infection of host cells by bacterial strains and also to quantify bacterial virulence. As shown in Fig. 2, the death rates of the THP-1 cells infected by S1, S8, and S9 were 1.25%–4.15%, 10.03%–16.30%, and 14.30%–30.32% (95% CI), respectively. The mortality caused by S9 was about 3.3–24.2 times that caused by S1, which means S9 is much more virulent than S1 (*P* < 0.0001). The virulence ranking would be S9 > S8 > S1, just the same as that obtained from the above SYTOX Green staining.

### Virulence test by survival of infected *Galleria mellonella* larvae

**Fig 3:**
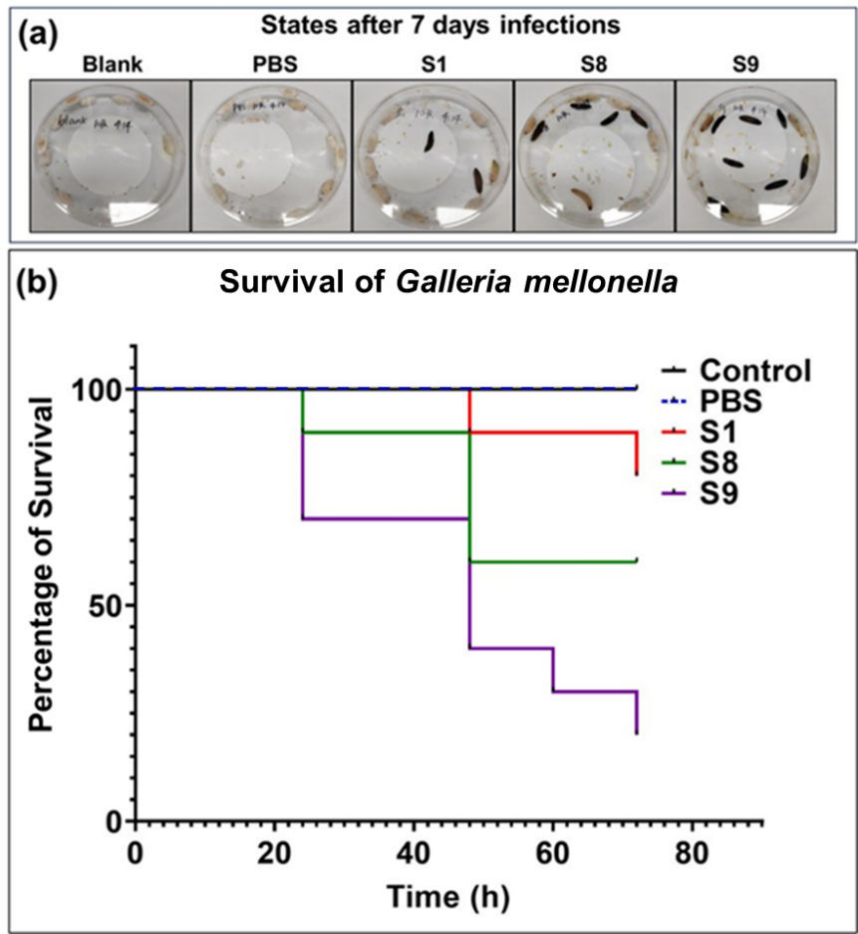
Survival curves of *G. mellonella* larvae infected with different bacterial strains or control. States of *G. mellonella* larvae in three groups (uninfected group, PBS-injected group, and groups infected with S1, S8, or S9) at 7 days post-treatment (a). Survival curves of *G. mellonella* larvae in the three groups (b).The ordinate of (b) is the percentage of survival.

Survival curves of infected *G. mellonella* larvae have been widely used to quantify *in vivo* bacterial virulence. In Fig. 3a, the gross view of the health status of the *G. mellonella* larvae uninfected, infected with phosphate-buffered saline (PBS), or S1, S8, and S9 after 7 days is presented. As can be seen, no death occurred for uninfected or injected with PBS, but 2, 4, and 7 dead larvae were seen in S1, S8, and S9 infected groups, respectively, resulting in a rank of virulence S9 > S8 > S1. In Fig. 3b, the 7-day survival curves of five groups (10 in each group) of *G. mellonella* larvae were plotted. The two control groups were *G. mellonella* larvae infected with no bacteria and with PBS. As can be seen, *G. mellonella* larvae infected by S9 died more rapidly than those infected by S1, and the 7-day survival rates of the *G. mellonella* larvae infected by S1, S8, and S9 were 80%, 60%, and 30%, respectively, which implies the virulence rank is also S9 > S8 > S1.

### Survival curves of mice infected with *S. muris* and *S. maltophilia* strains

**Fig 4:**
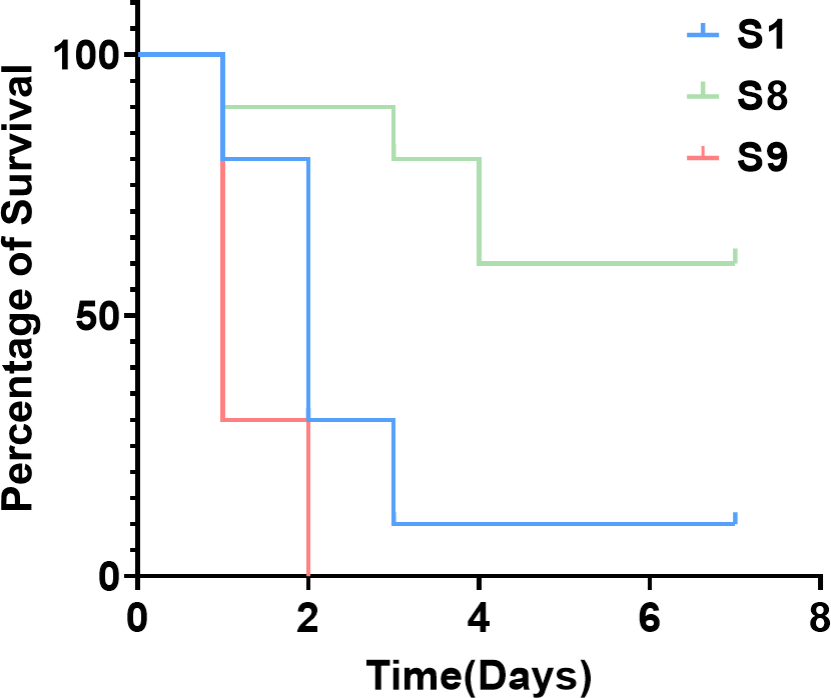
Survival of infected mice. In S1, S8, and S9 groups, the mice (10 per group) were infected by S1, S8, and S9 (with 1 × 10^8^ CFU per mouse), respectively, and observed for survival or death over 7 days. The ordinate is the percentage of survival.

Survival curves for mice infected with the *S. muris* and *S. maltophilia* strains were also employed because the mouse is a more relevant mammalian model. The survival curves of three groups (10 in each group) of mice (infected by S1, S8, and S9) are shown in Fig. 4. As can be seen in Fig. 4, S9 exhibited rather strong virulence because all mice in this group rapidly died within 2 days. The survival rates of the mice infected by S1, S8, and S9 were 10%, 60%, and 0%, respectively, again indicating that S9 is the most virulent.

### Whole-genome sequencing and average nucleotide identity analysis identified S9 and S8 as *S. muris* not *S. maltophilia*

**Fig 5:**
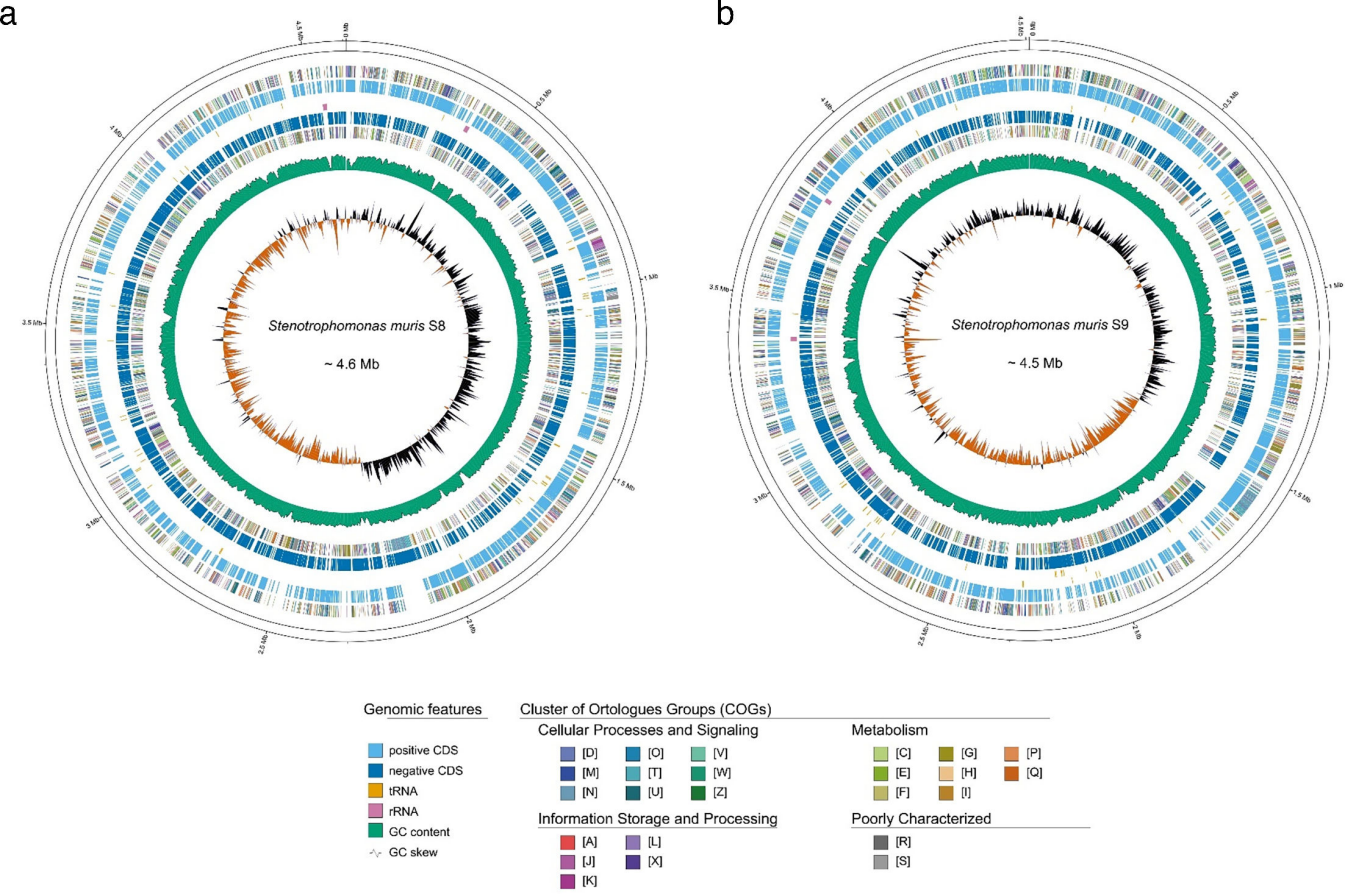
Circos plots of genomes of S8 (**a**) and S9 (**b**). Character COGs are as follows: D: cell cycle control, division, and chromosome partitioning; M: cell wall/membrane/envelope biogenesis; N: cell motility; O: post-translational modification, protein turnover, and chaperones; T: signal transduction mechanism; U: intracellular trafficking, secretion, and vesicular transport; V: defense mechanism; W: extracellular structures; Y: nuclear structure; Z: cytoskeleton; A: RNA processing and modification; B: chromatin structure and dynamics; J: translation, ribosomal structure, and biogenesis; K: transcription; L: replication, recombination, and repair; X: mobilome: prophages and transposons; C: energy production and conversion; E: amino acid transport and metabolism; F: nucleotide transport and metabolism; G: carbohydrate transport and metabolism; H: coenzyme transport and metabolism; I: lipid transport and metabolism; P: inorganic ion transport and metabolism; Q: secondary metabolites biosynthesis, transport, and metabolism; R: general function prediction only; S: function unknown.

WGS showed that the S8 genome is composed of 4,608,528 bp with the Guanine-Cytosine (GC) content of 66.8%, and there is no plasmid in the S8 genome. The whole genome of S9 contains a chromosome with 4,507,625 bp (66.8% GC) and a plasmid with 91,348 bp (67.7% GC). The circos plots of the genomes of S8 and S9 are shown in Fig. 5. The circos plot of the plasmid of S9 is shown in Fig. S1 in the Supplementary Materials. By analyzing the WGS data via the average nucleotide identity (ANI) method, it was shown that S8 and S9 were ~99% consistent with *S. muris*, but only ~92% consistent with *S. maltophilia*, which means both S8 and S9 should be more appropriately classified into this tentatively named species *S. muris*. Genomic sequence analyses further confirm that S8 and S9 are phylogenetically divergent from *S. maltophilia* and cluster with the taxon tentatively designated (see Supplementary Materials). Further analysis showed that there were 4,280 genes in S8 and 4,271 genes in S9 (4,197 genes in the chromosome and 74 genes in the plasmid).

### *In silico* analysis of virulence factors of S9 in the Virulence Factor Database

All S9 genes were compared with the Virulence Factor Database (VFDB), and 33 S9 genes (with an identity of >60%) were found in VFDB (Table S2). S8 genes and S1 genes annotated in VFDB are also listed (Table S2). Analysis of Table S2 shows that S8, S9, and S1 share 21 common genes, and about 71% of them are related to bacterial motility or pili, which are known to be associated with biofilm formation (6) and virulence (7). A previous study of 7 *S*. *maltophilia* clinical isolates found 106 shared strongly expressed genes associated with biofilm formation (6). By comparing our research findings with theirs, we found that S9 expresses several genes, including *flgG*, *flgI*, *fliG*, *fliM*, *fliP*, *flhA*, and *flhG*, from our study involved in flagellar synthesis and bacterial motility regulation. In addition, the *pilU*, *pilT*, *pilZ*, *pilM*, *pilG*, and *pilR* genes in our study are homologous to the *pilH* gene in the previous work (6). All these genes belong to the type IV pili-associated gene family and play roles in cell adhesion and biofilm formation. Compared with another study that explored the genetic differences between beneficial (*Stenotrophomonas rhizophila* DSM 14405) and pathogenic (*S. maltophilia* K279a) strains of *Stenotrophomonas* (8), we found that the virulence genes of S9 highly overlap with those of the pathogenic strain *S. maltophilia* K279a in terms of core functions such as motility, adhesion, secretion systems, stress tolerance, and drug resistance. This implies the potential of S9 as a human pathogen, and the same applies to S8.

In addition, four genes, including *algW*, *cheR*, *motA*, and *pilH*, should be taken into account, as they are in both S8 and S9 but not in S1. It was reported that *algW* in *P. aeruginosa* may play a role in biofilm formation (9), which may help the bacteria to better adapt to the living environment. *cheR* encodes methyltransferase (10), which helps the bacteria to find a better living environment, and *pilH* encodes a homolog of the ABC transporter (11), which may play a role in the multi-drug efflux pump. As a result, these three genes may all be related to the virulence of *S. muris*. Studies have shown that proteins encoded by *motA* can act synergistically with proteins encoded by *motB* to block ion channels in host cells (12), which may influence ion transportation of the host. It should be mentioned that genes in Table S2 are the *in silico* identified virulence genes and need further confirmation by gene knockout and animal studies in the future.

### Gene ontology and Kyoto Encyclopedia of Genes and Genomes enrichment analysis

We performed gene enrichment analysis with the gene ontology (GO) database and the Kyoto Encyclopedia of Genes and Genomes (KEGG) database. The target genes for enrichment were S9-unique genes, which were genes in S9 but not in S8. Since S1 is also less virulent than S9, these S9-unique genes vs S1 were excluded. There were 280 S9-unique genes, and 19 of them were in S1. In the remaining 261 S9-unique genes, 53 (listed in Table S1) were annotated in the GO database, and 14 (listed in Table 1) were annotated in the KEGG database.

**TABLE 1:** S9-unique genes annotated in the KEGG database and their pathways

Pathway	ID	Genes
DNA replication	ko03030	group_374
Porphyrin metabolism	ko00860	group_3203
Biofilm formation*—P. aeruginosa*	ko02025	group_3063
Pyrimidine metabolism	ko00240	group_832
Longevity regulating pathway—worm	ko04212	group_962
ABC transporters	ko02010	group_2016
Bacterial secretion system	ko03070	virB6
Beta-lactam resistance	ko01501	group_2745
Cell cycle—*Caulobacter*	ko04112	group_374; group_962
Exopolysaccharide biosynthesis	ko00543	group_3063; group_3011
Cysteine and methionine metabolism	ko00270	group_849; *dcm*
Host-pathogen interaction	ko04626	group_2016
Two-component system	ko02020	group_1035; group_1019

**Fig 6:**
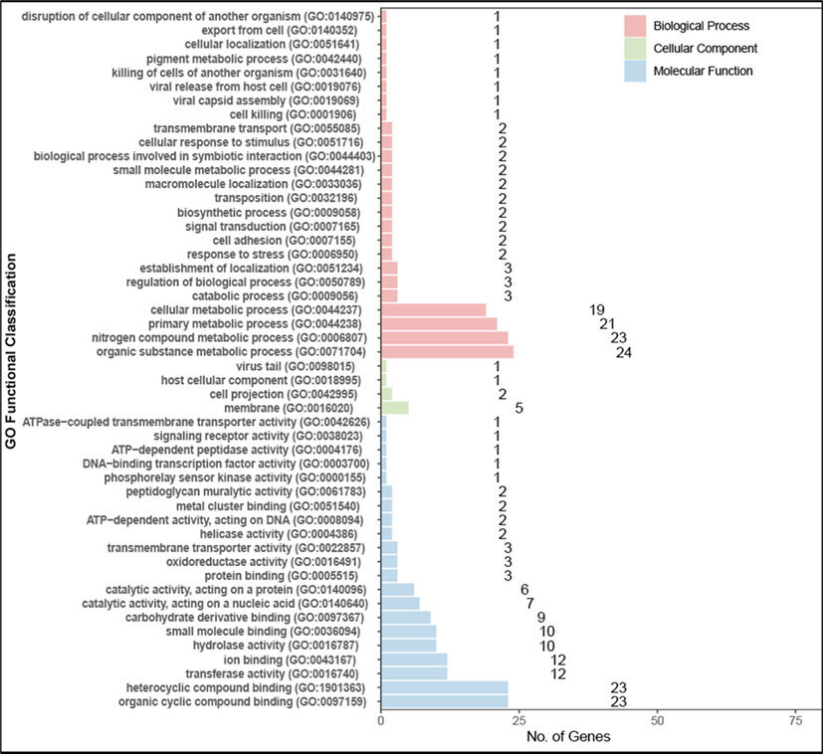
GO functional enrichment of S9-unique genes in S9- vs S8-infected THP-1 cells. In the figure, the items are grouped by three level 1 GO items: molecular function (MF, blue), cell component (CC, green), and biological process (BP, red).

The 53 GO-annotated S9-unique genes were enriched according to their gene functions (Fig. 6). The functions associated with more genes are regarded to be more likely to encode higher virulence, but only if these gene functions are related to bacterial virulence. As shown in Fig. 6, the top 10 (ranked by gene numbers) gene functions are cellular metabolic process, primary metabolic process, nitrogen compound metabolic process, organic substance metabolic process, small molecule binding, hydrolase activity, ion binding, transferase activity, heterocyclic compound binding, and organic cyclic compound binding. Among these, hydrolase activity and ion binding are most likely suspect, because some hydrolases, such as the esterase, are virulence factors in some bacteria (13–15), and the ion binding function may be associated with an efflux pump, involved in drug resistance in bacteria (16, 17). Transferase activity may contribute to the synthesis of many virulence factors. For example, the hyaluronic acid transferase of streptococci and the lipoteichoic acid transferase of staphylococci promote the synthesis of hyaluronic acid (18) and lipoteichoic acid (19), which are the virulence factors of the two bacteria, respectively. It was also reported that hyaluronic acid capsules may promote biofilm formation (20). Further studies are needed to confirm the role of these genes in the virulence of *S. muris*. KEGG enrichment analysis of 14 KEGG-annotated S9-unique genes and their associated pathways is listed in Table 1. Several pathways, including biofilm formation (21, 22), ABC transporters (23), bacterial secretion system (24), and two-component system (25, 26), need special attention because they are associated either with drug resistance or with bacterial virulence. In addition, the genes *virB6*, *dcm*, and *hlyD* also need special attention because *virB*6 is associated with sporty pili and type IV secretion system (virulence factors) of *Agrobacterium* (27), *dcm* is a DNA cytosine methylase gene that may affect the virulence and drug resistance of bacteria (28), and *hlyD* participates in secretion of hemolysin (a virulence factor) in *Escherichia coli* (29, 30). The comparison between GO enrichment and KEGG enrichment shows that the ion binding function and the ABC transporter pathway share the same gene group_2016, and therefore, the 10 genes associated with the ion binding function should be the candidate genes for future analysis.

### Host response to *S. muris* infection by transcriptome analysis

THP-1 cells infected by S1, S9, and S8 were subjected to RNA-seq analysis. Figure S3 presents statistics on the number of differentially expressed genes for S8 vs S1, S9 vs S1, and S9 vs S8. Among them, the numbers of upregulated genes are 78, 176, and 40, respectively, and the numbers of downregulated genes are 74, 226, and 13, respectively.

**Fig 7:**
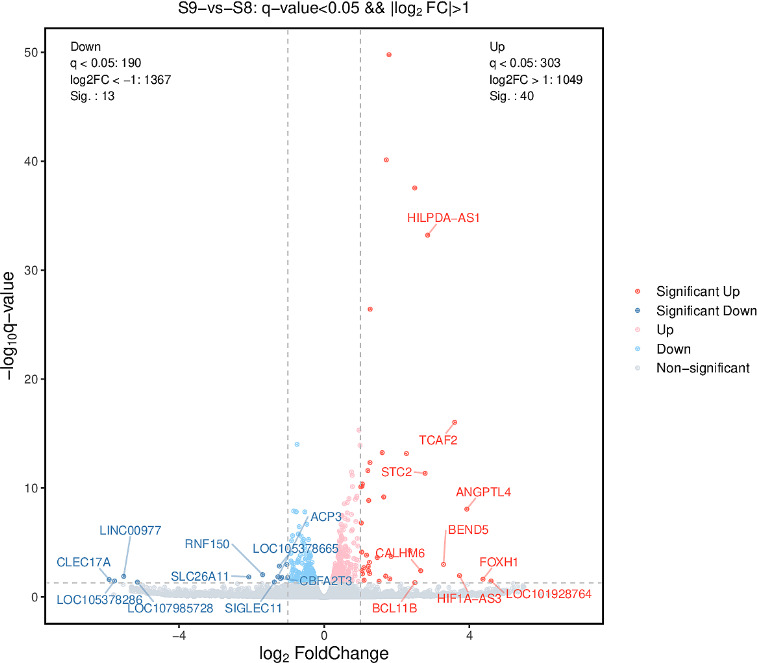
Volcano map of differentially expressed genes in S9- vs S8-infected THP-1 cells. The abscissa is the logarithm (2-based) of the difference multiple (Fold Change, FC), and the ordinate is the negative logarithm of the *q*-value. Gray points are differentially expressed genes below the threshold. Dark blue (points within the interval log_2_FC < −1 and −log_10_*q*-value > 1) and dark red (points within the interval log_2_FC > 1 and −log_10_*q*-value > 1) are significantly downregulated and upregulated genes, respectively. There are 40 upregulated genes and 13 downregulated genes.

**Fig 8:**
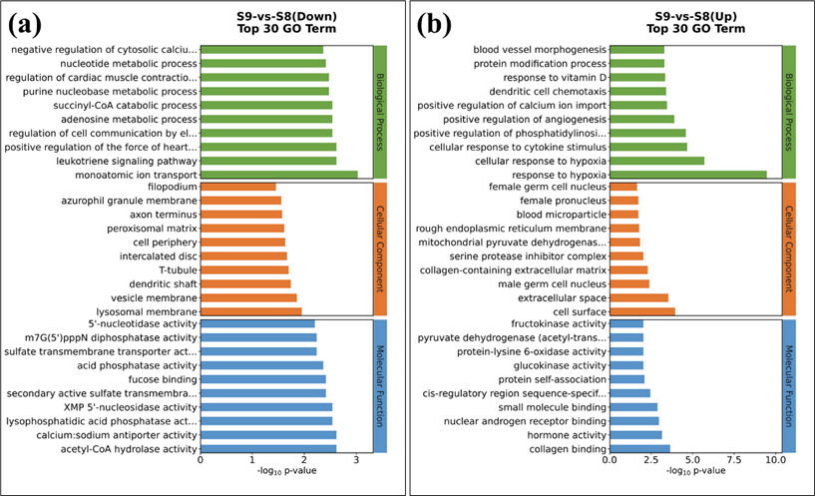
GO enrichment of S9 vs S8 differentially expressed genes in infected THP-1 cells. (**a**) Downregulated genes. (**b**) Upregulated genes. A larger log_10_*P*-value indicates a higher enrichment level.

Given that S9 and S8 are tentatively classified as conspecific, distinct from S1, and the core focus of this study is to elucidate the underlying mechanisms contributing to the enhanced virulence of S9, subsequent sections will primarily delve into the differential host transcriptomic profiles between S9 and S8, with S8 designated as the reference control. The differentially expressed genes in S9- vs S8-infected cells (i.e., host genes expressed in S9 but not in S8) are shown in Fig. 7. It was found that 40 genes were significantly upregulated and 13 significantly downregulated in S9-infected host cells, as are listed in Table S3. The GO enrichment analysis of these differentially expressed genes is shown in Fig. 8. The top 10 (ranked by -log_10_*P*-value) downregulated GO terms (gene functions) are monoatomic ion transport, leukotriene signaling pathway, positive regulation of the heart contraction, acetyl-CoA hydrolase activity, calcium:sodium antiporter activity, regulation of cell communication by electrical coupling, adenosine metabolic process, succinyl-CoA catabolic process, lysophosphatidic acid phosphatase activity, and XMP 5'-nucleosidase activity, and their corresponding genes are *SLC26A11*, *SLC8A1*, *CYSLTR1*, *NUDT7*, and *ACP3*. As discussed above, the ion-binding gene function should draw special attention because it may be associated with the higher virulence of S9. Here, it is found that monoatomic ion transport in S9-infected THP-1 cells is most seriously affected, which echoes the WGS result that the ion-binding gene function in S9 could be responsible for higher virulence. The two associated genes of monoatomic ion transport are *SLC26A11* and *SLC8A1*, where *SLC26A11* is related to the transport of chloride ion (31, 32), and *SLC8A1* is related to the transport of calcium (Ca²^+^) and sodium (Na^+^) ions (33), both of which are closely related to cellular respiration. These two positively charged ions remind us of the negatively charged (34) lipopolysaccharides (LPS), a component unique to the outer membrane of gram-negative bacteria, and exhibit toxicity for human cells (35). It has been reported that Na^+^ interacted non-specifically with LPS molecules by effectively shielding the negative charges of LPS, whereas Ca²^+^ engaged in specific interactions by cross-linking adjacent molecules within the monolayer (36). However, their interrelationship extends beyond this electrostatic complementarity. Notably, calcium and sodium ions, which are essential for maintaining bacterial membrane integrity, may indirectly influence LPS-mediated toxicity. For instance, Ca²^+^ could enhance enzymatic degradation efficiency through modulating LPS aggregation states (37). Since LPS may act as a factor affecting ion transport of the host, it should be suspected to be a virulence factor that causes the higher virulence of S9, but needs further experimental verification.

The top two upregulated GO terms are response to hypoxia and cellular response to hypoxia, which involved 12 genes, including *HILPDA*, *ANGPTL4*, *SLC2A1*, *HK2*, *ADM*, *CXCR4*, *BNIP3*, *PLAT*, *PLOD2*, *STC2*, *STC1*, and *AK4*. *SLC26A11* could be the major chloride entry pathway under hypoxia (31). Thus, we can form a hypothesis: infection of S9 hindered the oxygen intake of THP-1 cells, then the cells generated a stress response, leading to the upregulation of corresponding gene functions. After the resolution of hypoxia, the chloride entry pathways under hypoxic conditions were downregulated. *BNIP3* should also be specially mentioned because its upregulation can promote the release of apoptosis factors (38). Detailed information (gene functions) of the 12 involved genes is listed in Table 2. In Table 2, the most upregulated gene *FOXH1* is also listed, as well as its gene functions. Two previous studies have reported the transcriptome analyses of host cells infected by *S. maltophilia* (39, 40). By comparing the transcriptome analysis results of the present study, we found that there are differences between the host response to S9 infection and its response to *S. maltophilia* infection, indicating that S9 may have a pathogenic mechanism different from both of the reported ones.

**TABLE 2:** Functions of significantly upregulated genes in cellular response to S9 vs S8 infection in THP-1

Gene name	Gene functions
*HILPDA*	Hypoxia inducible lipid droplet associated (HILPDA) involved in lipid metabolism (41)
*ANGPTL4*	Lipid metabolism regulation, glucose metabolism, and tumor regulation (42)
*SLC2A1*	Glucose transport and cell energy balance (43)
*HK2*	Glycolysis initiation and tumor cell energy supply (44)
*ADM*	Vasodilation, blood pressure regulation, and cell process regulation (45)
*CXCR4*	Cell chemotaxis, immune cell homing, and tumor metastasis (46)
*BNIP3*	Cell apoptosis induction and autophagy regulation (47)
*PLAT*	Fibrinolytic activation (48)
*PLOD2*	Collagen hydroxylation and tumor extracellular matrix remodeling (49)
*STC2*	Cell process regulation and calcium and phosphorus metabolism (50)
*STC1*	Calcium and phosphorus metabolism regulation (50) and tissue development (51)
*AK4*	Intracellular energy metabolism and signal transduction (52)
*FOXH1*	Congenital disease (53, 54), tumor proliferation, migration, and invasion (55, 56)

**Fig 9:**
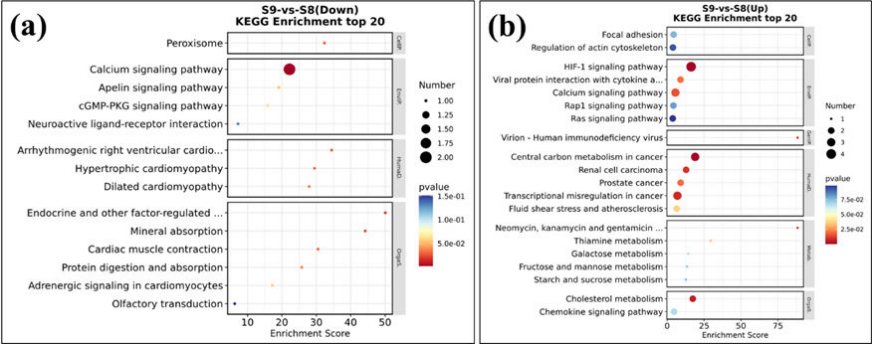
KEGG enrichment of S9 vs S8 differentially expressed genes in infected THP-1 cells. (**a**) Downregulated genes. (**b**) Upregulated genes. Smaller *P*-values and larger enrichment scores indicate a higher enrichment level.

The KEGG enrichment analysis also supports the above findings (see Fig. 9). In the downregulated genes, the calcium signaling pathway has the highest enrichment level, a finding that is consistent with one of the associated genes being *SLC8A1*, which is related to the transport of calcium and sodium ions (33). In the upregulated genes, the HIF-1 signaling pathway is at the highest enrichment level. HIF-1, the hypoxia-inducible factor 1, is a helix-loop-helix transcription factor that can activate genes involved in hypoxic homeostasis response proteins (57), which again indicates that infection of S9 may affect the oxygen intake of THP-1 cells.

### Antibiotic susceptibility testing

To determine potential treatment response of the *S. muris* strains, we also determined their antibiotic susceptibility to six commonly used antibiotics, including ceftazidime, levofloxacin, colistin, polymyxin B, sulfamethoxazole/trimethoprim, minocycline, and ceftazidime for treatment of *S. maltophilia* infections (1). The results of the antibiotic susceptibility testing for S1, S8, and S9 are listed in Table 3. The resistance clinical breakpoints of all drugs, except colistin, for *S. maltophilia* referred to the values suggested by the CLSI, and the resistance clinical breakpoint of colistin for *S. maltophilia* referred to the value suggested by the European Committee on Antimicrobial Susceptibility Testing (EUCAST, 2018) for *Pseudomonas sp.* (58).

**TABLE 3:** Results of antibiotic susceptibility testing for strains S1, S8, and S9^*a*^

Antibiotics	S1		S8		S9	
	MIC	Susceptibility	MIC	Susceptibility	MIC	Susceptibility
Ceftazidime	0.125	S	2	S	4	S
Levofloxacin	<0.06	S	0.5	S	2	I
Colistin	2	S	64	R	128	R
Polymyxin B	2	S	32	R	64	R
Sulfamethoxazole/trimethoprim	8	R	8	R	8	R
Minocycline	0.125	S	<0.06	S	<0.06	S

^*a*^ The MICs (μg/mL) and susceptibility (susc) of different antibiotics for *S. maltophilia* S1, *S. Muris* S8, and S9 are listed. “S” means susceptible, “I” means intermediate, and “R” means resistant.

As shown in Table 3, S8 and S9 are highly resistant to colistin and polymyxin B, with their MIC values exceeding the established resistance breakpoints. For ceftazidime (a cephalosporin), while the MIC values in S8 and S9 were higher than S1, they remained within the susceptible range based on the breakpoints. A similar trend was seen with levofloxacin (a fluoroquinolone): though their MIC values were elevated relative to S1, S8 stayed in the susceptible category, and S9 fell into the intermediate resistance category. Thus, S8 and S9 exhibited increased—but not necessarily resistant—MIC values for ceftazidime and levofloxacin. However, it is of interest to note that both S8 and S9 were quite sensitive to minocycline (MIC <0.06 µg/mL), which may be a specific and useful medication for the treatment of *S. muris* infections. Further studies are needed to confirm these findings in the clinic. Furthermore, an epidemiological study is needed to determine if there is indeed a difference between these two genomospecies.

### Conclusions

Although *S. muris* has not yet been published in the updated taxonomy list of IJSEM, as the description in the original article (59) did not meet all criteria for type strain deposition in two different countries’ culture collections, the lack of official recognition should not negate the biological distinctiveness of this organism from *S. maltophilia*. To substantiate this distinction, more analyses with comprehensive genomic evidence are presented to show that strains S8 and S9 share ANI values of 98.54% and 98.70% with *S. muris* DSM28631, while only 92.44% and 92.47% with *S. maltophilia* ATCC13637, well below the 96% threshold for species designation. In fact, this is reflected in a recent paper (60). In this paper, many related *S. maltophilia* species that have ANI values below 95% have not yet been recognized as official species. Phylogenetic analyses based on both 16S rRNA sequences and whole-genome sequence data (Fig. S2) further confirm the evolutionary relationship between S8/S9 and *S. muris*.

In summary, *S. muris* clinical isolates were found to have high virulence. The *S. muris s*train S9 isolated from the patient’s bloodstream infection has stronger virulence than the pulmonary isolate S8 and *S. maltophilia* type strain, which are associated with unique candidate genes that may encode the higher virulence, including *virB6*, *dcm*, *hlyD*, and 14 other genes of unknown function. RNA-seq analysis suggests that the highly virulent *S. muris* strain S9 may preferentially hinder the oxygen intake, ion transport, and calcium signaling of THP-1 cells, and LPS, which may affect ion transport of the host, should be suspected as a virulence factor of S9. Antibiotic susceptibility testing indicated that compared with *S. maltophilia*, the *S. muris* strains, though more susceptible to minocycline, were highly resistant to last resort antibiotics colistin and polymyxin B and were also resistant to cephalosporin and fluoroquinolone antibiotics, which may need to be taken into consideration when treating *S. muris* infections. Because of the above differences in virulence properties and antibiotic susceptibility, it is critical that *S. muris* be distinguished from *S. maltophilia* for clinical surveillance and for improved treatment outcomes in the future.

## MATERIALS AND METHODS

### Source of bacterial strains and clinical isolates S8 and S9

S1 was *S. maltophilia* type strain from ATCC. S8 was isolated from the sputum of a male patient in his 60 s, diagnosed with acute lymphoblastic leukemia accompanied by hemorrhagic pneumonia. The antibiotic treatment was not effective, and the patient had a poor outcome. S9 was isolated from the blood of a male patient in his 20 s, diagnosed with septic shock, severe pneumonia, disseminated mucormycosis, and multiple organ failure. S8 and S9 were initially identified as *S. maltophilia* by standard routine MALDI-TOF mass spectrometry (Vitek MS system, bioMerieux, France).

### SYTOX Green staining

THP-1 cells were seeded into a 12-well tissue culture plate at a density of 5 × 10^5^ cells per well. The 12 wells were divided into four groups with three wells in each group, and group 1 was the control group with THP-1 cells infected by no bacteria. In groups 2–4, the cells were infected by S1, S8, and S9 with the multiplicity of infection (MOI = 1), respectively. All cells were incubated at 37°C for 18 (or 8) h and then washed by Hanks’ buffered salt solution. Then the washed cells were stained with SYTOX Green (Thermo Fisher Scientific) for 30 minutes. Several images were taken by a fluorescence microscope with 10× magnification for each well, and the images with the best quality were chosen. The ranking of staining level was based on visual assessment during direct microscopic examination. These experiments were repeated three times to confirm the findings.

### LDH release assay

Six groups of THP-1 cells were seeded into a 96-well plate, and each group had six replicates. Three control groups were treated as follows: the first group, which was named the blank group, had only culture medium; the second group, named the sample group, had both culture medium and uninfected cells; the last group, named the max group, also had both culture medium and uninfected cells. However, the cells were completely lysed after culture. Three experimental groups were infected by S1, S8, and S9 (with the MOI = 1:1), respectively. After a 23 h incubation at 37°C, the reagent used to lyse cells was added to the max group, and then incubation was continued. After 24 h, incubation of all groups was stopped. Then THP-1 cells were centrifuged for 5 minutes, and the supernatants were transferred to a new 96-well plate. Then, the LDH solution was added to each well and incubated at 37°C for 30 minutes in the dark. Afterward, absorbance at 490 nm was measured for each well in a Biotek plate reader. The absorbance of the blank group was defined as *A*_0_, the absorbance of the sample group as *A*_*s*_, the absorbance of the max group as *A*_*m*_, and the absorbance of the experiment group as *A*_*e*_, then the death rate was calculated as *M*_cell_ (%) = (*A*_*e*_ − *A*_*c*_ − *A*_0_)/(*A*_*m*_ − *A*_*c*_ − *A*_0_) × 100% . Generally, *A*_0_ is quite smaller than *A*_*c*_, so the formula can be simplified as *M*_cell_ (%) = (*A*_*e*_ − *A*_*c*_)/(*A*_*m*_ − *A*_*c*_) × 100%. After obtaining the absorbances of each well, there were six *A*_0_s and six *A*_*c*_s. Then the averaged *A*_0_ and *A*_*c*_ were calculated by averaging all *A*_0_s and *A*_*c*_s. Finally, the death rates for all wells in the experiment groups (with the help of the averaged *A*_*c*_) were calculated, and GraphPad was used to analyze the data for S1, S8, and S9. The LDH assay was performed according to the instructions of the LDH kit (Beyotime Biotechnology, Shanghai, China).

### Survival curves of *G. mellonella*

Five groups (10 in each group) of *G. mellonella* larvae with similar health conditions were used. Two groups were the control groups without any bacterial infections, and the other three groups were infected by S1, S8, and S9, respectively. Dead or alive states were recorded daily until the scheduled time. The experiments were stopped after 72 h. Bacteria were prepared as follows. A single colony was inoculated into Luria-Bertani (LB) broth and incubated at 37°C for 20 h with shaking. The overnight culture was centrifuged, and the pelleted bacteria were resuspended in PBS at a concentration of 1 × 10^8^ CFU/mL. *G. mellonella* larvae were infected by injection of 10 µL of the bacterial suspension (with the final inoculum size 1 × 10^6^ CFU per *G. mellonella* larva). One control group was not treated with any drugs or culture medium, and the other control group was injected with 10 µL of PBS.

### Survival curves of infected mice

Female Balb/c mice aged 6–8 weeks were used in this experiment. The immune functions of all mice are normal. Bacterial inocula were prepared with the same procedures as those used in the *G. mellonella* larvae test. Three groups (10 in each group) of mice were infected via nasal instillation with 50 µL of suspensions (final inoculum size of 1 × 10^8^ CFU per mouse) of S1, S8, and S9, respectively. All mice were fed in the same environment with *ad libitum* access to food and water. Dead or alive states of infected mice were recorded daily for 7 days.

### Whole-genome sequencing

AxyPrep bacterial genomic DNA miniprep kit (Axygen Scientific, Union City, CA, USA) was used to extract DNA of the target bacteria. The Illumina HiSeq 2500 platform (paired-end run; 2 × 150 bp) together with an Oxford Nanopore MinION platform was employed to perform the whole-genome sequencing. The Assembly and SRA databases of NCBI were utilized to obtain the publicly available genome assemblies and short-read data for S1, S8, and S9. Kingfisher v7.6.1 (https://github.com/onevcat/Kingfisher) and ncbi-genome-download v0.3.1 (https://github.com/kblin/ncbi-genome-download) were used during the data retrieval. Fastp v0.23.2 was used to filter qualities and trim adaptors of sequencing reads. Careful-mode shovill v1.1.0 (https://github.com/tseemann/shovill) was used to assemble trimmed reads and contigs that possess less than 200 bp. With the help of the Unicycler hybrid assembly pipeline, long-read data from Oxford Nanopore MinION sequencing and short-read data from Illumina sequencing were synergized to generate the complete genomes of S1, S8, and S9. ANI was utilized to identify species of S8 and S9.

### RNA-seq analysis of infected host cells

Six groups of THP-1 cells infected by S8 and S9 were prepared. S8, which had weak virulence, served as a control for more virulent S9 in RNA-seq analysis. Three groups (S8-1, S8-2, and S8-3) were infected by S8, and three groups (S9-1, S9-2, and S9-3) were infected by S9 (the MOIs were all 1:1, and the infection time was 24 h). The total RNAs of these six groups of THP-1 cells (after infections) were extracted using the total RNA Trizol kit (Beyotime Biotechnology, Shanghai, China). Before sequencing, rRNAs were removed from the total RNAs, then the remaining RNAs were converted into cDNAs by reverse transcription. The sequencing methods of cDNAs were the same as DNAs in WGS. The accession number for the RNA-Seq data is PRJNA1243718.

After getting the DNA sequencing reads, they were aligned to the reference genome to obtain the genome alignment status of each sample. Then the expression levels of these protein-coding genes were analyzed based on the alignment results. Afterward, these genes were divided into two groups: one is the S8 group that contains all S8-infected cells, and the other is the S9 group that contains all S8-infected cells. Then the differentially expressed genes of the two groups were screened. Finally, enrichment analyses of the differentially expressed genes were performed based on the GO and KEGG databases to obtain the gene functions, signaling pathways of the differentially expressed genes, along with their up- and downregulation status.

### Antibiotic susceptibility testing

The standard microdilution method was used to determine the MIC. *S. maltophilia* ATCC 25922 (S1), *S. muris* S8, and S9 were cultured in LB broth with shaking overnight at 37°C. The bacterial suspension was adjusted to 0.5 McFarland and then diluted 1:100 in CAMHB medium. Then, 100 µL diluted culture was transferred to 96-well microtiter plates and mixed with serial twofold dilutions of ceftazidime, levofloxacin, colistin sulfate, polymyxin B, minocycline, and sulfamethoxazole/trimethoprim (5:1) in concentrations ranging from 128 μg/mL to 0.06 μg/mL by the microdilution method. The negative control contained only CAMHB, and the positive control contained diluted culture in CAMHB. Assay plates were incubated without shaking at 37 °C for 20 h. The MIC was the lowest concentration of each drug where no visible growth was seen in the wells. All experiments were run in triplicate.

## Data Availability

Genome sequences of strains S8 and S9 are available in NCBI under BioProject no. PRJNA1226979.

## SUPPLEMENTAL MATERIAL

Supplemental Material spectrum.02770-24-s0001.docx Tables S1 to S3; Fig. S1 to S3.

ASM does not own the copyrights to Supplemental Material that may be linked to, or accessed through, an article. The authors have granted ASM a non-exclusive, world-wide license to publish the Supplemental Material files. Please contact the corresponding author directly for reuse.
