# Supplementary material for: Stenotrophomonas muris—first discovered as a potential human pathogen with strong virulence and antibiotic resistance, associated with bloodstream infections
Source: Microbiol Spectr. 2025 Sep 23;13(11):e02770-24. doi: 10.1128/spectrum.02770-24 (PMC12584626; doi:10.1128/spectrum.02770-24)
Supplement: Supplemental Material — Tables S1 to S3; Fig. S1 to S3. [file spectrum.02770-24-s0001.docx]

**Table of Figure Legends for supplementary figures**

| Figure S1 | **Circos plot of genome of the plasmid in S9.** |
| --- | --- |
| Figure S2 | **Evolutionary relationships between** **S8/S9 and** ***S. muris*, which act as supplementary evidence that confirms S8 and S9 as *S. muris*.** |
| Figure S3 | **Numbers of differentially expressed genes in S8 versus S1, S9 versus S1 and S9 versus S8 infected cells.** |

**TABLE S1** S9-unique genes in GO database. All the genes are only in S9 strain. They were obtained by getting S9 genes different from S8 and then eliminating S1-contained genes from the obtained genes.

| Gene_ID | GO_term | Function_class | Function |
| --- | --- | --- | --- |
| dcm_orf5049 | GO:0003824 | molecular_function | catalytic activity |
| group_1014_orf4779 | GO:0003824 | molecular_function | catalytic activity |
| group_1019_orf4993 | GO:0009987 | biological_process | cellular process |
| group_1019_orf4993 | GO:0110165 | cellular_component | cellular anatomical entity |
| group_1035_orf4930 | GO:0009987 | biological_process | cellular process |
| group_1035_orf4930 | GO:0110165 | cellular_component | cellular anatomical entity |
| group_1077_orf5011 | GO:0003824 | molecular_function | catalytic activity |
| group_1165_orf1487 | GO:0003824 | molecular_function | catalytic activity |
| group_1165_orf1487 | GO:0005488 | molecular_function | binding |
| group_1165_orf1487 | GO:0008152 | biological_process | metabolic process |
| group_1165_orf1487 | GO:0009987 | biological_process | cellular process |
| group_1239_orf1295 | GO:0003824 | molecular_function | catalytic activity |
| group_1239_orf1295 | GO:0005488 | molecular_function | binding |
| group_1239_orf1295 | GO:0008152 | biological_process | metabolic process |
| group_1239_orf1295 | GO:0009987 | biological_process | cellular process |
| group_1285_orf647 | GO:0003824 | molecular_function | catalytic activity |
| group_1285_orf647 | GO:0005488 | molecular_function | binding |
| group_1286_orf1369 | GO:0003824 | molecular_function | catalytic activity |
| group_1286_orf1369 | GO:0005488 | molecular_function | binding |
| group_1286_orf1369 | GO:0008152 | biological_process | metabolic process |
| group_1286_orf1369 | GO:0009987 | biological_process | cellular process |
| group_1286_orf1369 | GO:0140657 | molecular_function | ATP-dependent activity |
| group_1317_orf1396 | GO:0003824 | molecular_function | catalytic activity |
| group_1317_orf1396 | GO:0005488 | molecular_function | binding |
| group_1317_orf1396 | GO:0008152 | biological_process | metabolic process |
| group_1317_orf1397 | GO:0005488 | molecular_function | binding |
| group_1345_orf1100 | GO:0016032 | biological_process | viral process |
| group_1352_orf1175 | GO:0003824 | molecular_function | catalytic activity |
| group_1352_orf1175 | GO:0005488 | molecular_function | binding |
| group_1352_orf1175 | GO:0008152 | biological_process | metabolic process |
| group_1352_orf1175 | GO:0009987 | biological_process | cellular process |
| group_1383_orf1410 | GO:0005488 | molecular_function | binding |
| group_1383_orf1410 | GO:0008152 | biological_process | metabolic process |
| group_1383_orf1410 | GO:0009987 | biological_process | cellular process |
| group_1383_orf1410 | GO:0050896 | biological_process | response to stimulus |
| group_1462_orf536 | GO:0065007 | biological_process | biological regulation |
| group_1462_orf536 | GO:0140110 | molecular_function | transcription regulator activity |
| group_1757_orf1785 | GO:0003824 | molecular_function | catalytic activity |
| group_1757_orf1785 | GO:0008152 | biological_process | metabolic process |
| group_1757_orf1785 | GO:0009987 | biological_process | cellular process |
| group_1757_orf1785 | GO:0065007 | biological_process | biological regulation |
| group_1757_orf1785 | GO:0140299 | molecular_function | small molecule sensor activity |
| group_1966_orf4519 | GO:0005488 | molecular_function | binding |
| group_1966_orf4519 | GO:0008152 | biological_process | metabolic process |
| group_1966_orf4519 | GO:0009987 | biological_process | cellular process |
| group_1966_orf4520 | GO:0005488 | molecular_function | binding |
| group_1966_orf4520 | GO:0008152 | biological_process | metabolic process |
| group_1966_orf4520 | GO:0009987 | biological_process | cellular process |
| group_2016_orf3846 | GO:0003824 | molecular_function | catalytic activity |
| group_2016_orf3846 | GO:0005215 | molecular_function | transporter activity |
| group_2016_orf3846 | GO:0005488 | molecular_function | binding |
| group_2016_orf3846 | GO:0008152 | biological_process | metabolic process |
| group_2016_orf3846 | GO:0009987 | biological_process | cellular process |
| group_2016_orf3846 | GO:0051179 | biological_process | localization |
| group_2016_orf3846 | GO:0110165 | cellular_component | cellular anatomical entity |
| group_2016_orf3846 | GO:0140657 | molecular_function | ATP-dependent activity |
| group_2148_orf3752 | GO:0008152 | biological_process | metabolic process |
| group_2259_orf3945 | GO:0003824 | molecular_function | catalytic activity |
| group_2259_orf3945 | GO:0005488 | molecular_function | binding |
| group_2355_orf4489 | GO:0005488 | molecular_function | binding |
| group_2361_orf4077 | GO:0005488 | molecular_function | binding |
| group_2361_orf4077 | GO:0008152 | biological_process | metabolic process |
| group_2361_orf4077 | GO:0009987 | biological_process | cellular process |
| group_2368_orf4088 | GO:0003824 | molecular_function | catalytic activity |
| group_2368_orf4088 | GO:0005488 | molecular_function | binding |
| group_2368_orf4088 | GO:0008152 | biological_process | metabolic process |
| group_2368_orf4088 | GO:0009987 | biological_process | cellular process |
| group_2745_orf4196 | GO:0005215 | molecular_function | transporter activity |
| group_2745_orf4196 | GO:0009987 | biological_process | cellular process |
| group_2745_orf4196 | GO:0051179 | biological_process | localization |
| group_2745_orf4196 | GO:0110165 | cellular_component | cellular anatomical entity |
| group_2810_orf4237 | GO:0003824 | molecular_function | catalytic activity |
| group_2810_orf4237 | GO:0005488 | molecular_function | binding |
| group_2810_orf4237 | GO:0008152 | biological_process | metabolic process |
| group_2810_orf4237 | GO:0009987 | biological_process | cellular process |
| group_2942_orf2107 | GO:0005215 | molecular_function | transporter activity |
| group_2942_orf2107 | GO:0110165 | cellular_component | cellular anatomical entity |
| group_3011_orf2123 | GO:0003824 | molecular_function | catalytic activity |
| group_3203_orf1996 | GO:0003824 | molecular_function | catalytic activity |
| group_3203_orf1996 | GO:0008152 | biological_process | metabolic process |
| group_3203_orf1996 | GO:0009987 | biological_process | cellular process |
| group_342_orf1584 | GO:0008152 | biological_process | metabolic process |
| group_342_orf1584 | GO:0009987 | biological_process | cellular process |
| group_342_orf1584 | GO:0065007 | biological_process | biological regulation |
| group_347_orf1673 | GO:0003824 | molecular_function | catalytic activity |
| group_347_orf1673 | GO:0005488 | molecular_function | binding |
| group_374_orf104 | GO:0003824 | molecular_function | catalytic activity |
| group_374_orf104 | GO:0005488 | molecular_function | binding |
| group_374_orf104 | GO:0008152 | biological_process | metabolic process |
| group_374_orf104 | GO:0009987 | biological_process | cellular process |
| group_374_orf104 | GO:0140657 | molecular_function | ATP-dependent activity |
| group_603_orf15 | GO:0005488 | molecular_function | binding |
| group_603_orf15 | GO:0008152 | biological_process | metabolic process |
| group_603_orf15 | GO:0009987 | biological_process | cellular process |
| group_634_orf1618 | GO:0003824 | molecular_function | catalytic activity |
| group_634_orf1618 | GO:0005488 | molecular_function | binding |
| group_634_orf1618 | GO:0008152 | biological_process | metabolic process |
| group_634_orf1618 | GO:0009987 | biological_process | cellular process |
| group_832_orf2454 | GO:0003824 | molecular_function | catalytic activity |
| group_832_orf2454 | GO:0008152 | biological_process | metabolic process |
| group_832_orf2454 | GO:0009987 | biological_process | cellular process |
| group_835_orf3533 | GO:0005488 | molecular_function | binding |
| group_835_orf3533 | GO:0060089 | molecular_function | molecular transducer activity |
| group_840_orf2544 | GO:0003824 | molecular_function | catalytic activity |
| group_840_orf2544 | GO:0005488 | molecular_function | binding |
| group_840_orf2544 | GO:0008152 | biological_process | metabolic process |
| group_840_orf2544 | GO:0009987 | biological_process | cellular process |
| group_849_orf2314 | GO:0003824 | molecular_function | catalytic activity |
| group_857_orf3358 | GO:0005488 | molecular_function | binding |
| group_857_orf3358 | GO:0044419 | biological_process | biological process involved in interspecies interaction between organisms |
| group_857_orf3358 | GO:0044423 | cellular_component | virion component |
| group_857_orf3358 | GO:0110165 | cellular_component | cellular anatomical entity |
| group_873_orf3634 | GO:0005488 | molecular_function | binding |
| group_879_orf2616 | GO:0003824 | molecular_function | catalytic activity |
| group_879_orf2616 | GO:0005488 | molecular_function | binding |
| group_911_orf2666 | GO:0003824 | molecular_function | catalytic activity |
| group_911_orf2666 | GO:0005488 | molecular_function | binding |
| group_926_orf2951 | GO:0003824 | molecular_function | catalytic activity |
| group_926_orf2951 | GO:0008152 | biological_process | metabolic process |
| group_926_orf2951 | GO:0009987 | biological_process | cellular process |
| group_926_orf2951 | GO:0016032 | biological_process | viral process |
| group_926_orf2951 | GO:0044419 | biological_process | biological process involved in interspecies interaction between organisms |
| group_943_orf3011 | GO:0003824 | molecular_function | catalytic activity |
| group_943_orf3011 | GO:0005488 | molecular_function | binding |
| group_957_orf3376 | GO:0003824 | molecular_function | catalytic activity |
| group_957_orf3376 | GO:0005488 | molecular_function | binding |
| group_962_orf3117 | GO:0003824 | molecular_function | catalytic activity |
| group_962_orf3117 | GO:0008152 | biological_process | metabolic process |
| group_962_orf3117 | GO:0140657 | molecular_function | ATP-dependent activity |
| group_966_orf2674 | GO:0005488 | molecular_function | binding |
| group_966_orf2674 | GO:0008152 | biological_process | metabolic process |
| group_966_orf2674 | GO:0009987 | biological_process | cellular process |
| group_986_orf2975 | GO:0009987 | biological_process | cellular process |
| group_986_orf2975 | GO:0016032 | biological_process | viral process |
| group_986_orf2975 | GO:0044419 | biological_process | biological process involved in interspecies interaction between organisms |
| higA_orf1544 | GO:0005488 | molecular_function | binding |
| hlyD_orf3802 | GO:0009987 | biological_process | cellular process |
| hlyD_orf3802 | GO:0051179 | biological_process | localization |
| hlyD_orf3802 | GO:0110165 | cellular_component | cellular anatomical entity |
| sRAP_orf4636 | GO:0005488 | molecular_function | binding |
| sRAP_orf4636 | GO:0008152 | biological_process | metabolic process |
| sRAP_orf4636 | GO:0009987 | biological_process | cellular process |
| sRAP_orf4636 | GO:0050896 | biological_process | response to stimulus |
| trbL_orf4064 | GO:0009987 | biological_process | cellular process |
| trbL_orf4064 | GO:0051179 | biological_process | localization |
| virB6_orf4048 | GO:0009987 | biological_process | cellular process |
| virB6_orf4048 | GO:0051179 | biological_process | localization |

**TABLE S2** Putative virulence genes of S1, S8 and S9 in VFDB database (with identity > 60%)

| S1 genes | Identity (%) | S8 genes | Identity (%) | S9 genes | Identity (%) |
| --- | --- | --- | --- | --- | --- |
| PA3349 | 65.55 | htpB | 64.62 | nueA | 67.82 |
| flgG | 69.12 | acrB | 68.98 | katA | 76.22 |
| flgI | 71.49 | waaA | 67 | pilU | 77.62 |
| fliG | 69.09 | flmH | 69.96 | pilT | 80.37 |
| fliM | 74.83 | acpXL | 74.07 | pilZ | 72.37 |
| fliP | 71.84 | pilZ | 71.77 | acpXL | 74.07 |
| flhA | 72.02 | tapT | 74.82 | flmH | 69.65 |
| flhG | 70.85 | pilU | 77.81 | cheW | 75.94 |
| cheY | 76.69 | katA | 76.37 | motC | 70.58 |
| motC | 69.31 | nueA | 67.67 | cheY | 77.81 |
| cheW | 75.06 | kdsA | 65.55 | flhG | 71 |
| cheB | 66.7 | motA | 67.95 | flhA | 71.89 |
| xcpA/pilD | 68.06 | algA | 69.31 | fliP | 73.16 |
| tapC | 65.82 | rfbK1 | 66.19 | fliM | 74.46 |
| pilR | 71.06 | flgG | 69.79 | fliG | 69.73 |
| pilG | 79.53 | flgI | 70.9 | flgI | 71.08 |
| flmH | 69.65 | icl | 73.76 | flgG | 69.92 |
| acpXL | 74.07 | algW | 65.04 | pilM | 68.31 |
| pilZ | 72.67 | pilR | 72.2 | motA | 67.95 |
| pilT | 79.3 | tapC | 65.66 | algA | 69.38 |
| pilU | 77.13 | xcpA/pilD | 68.64 | rfbK1 | 66.19 |
| katA | 75.84 | pilM | 68.61 | cheB | 67.8 |
| acrB | 68.87 | cheB | 67.63 | cheR | 68.09 |
| htpB | 64.71 | cheR | 67.7 | acrB | 69.11 |
| rfbK1 | 68.43 | xcpT | 69.92 | htpB | 64.69 |
| algA | 68.55 | pilG | 80.42 | Icl | 74.25 |
| narH | 71.42 | pilH | 69.38 | algW | 65.04 |
| xcpT | 70.49 | fliG | 69.68 | pilH | 70 |
| tufA | 67.61 | fliM | 74.46 | waaA | 67 |
| tufA | 67.73 | fliP | 72.83 | pilR | 71.84 |
| pilM | 69.67 | flhA | 71.67 | xcpA/pilD | 68.77 |
| icl | 74.61 | flhG | 71.17 | pilG | 80.42 |
| pchD | 68.41 | cheY | 77.53 |  |  |
| waaA | 66.19 | motC | 70.74 |  |  |
|  |  | cheW | 75.69 |  |  |

**TABLE S3** Significantly differentially expressed genes for S9-vs-S8. Here, S9 and S8 are THP-1 cells infected by S9 and S8, respectively. The log2FC describes the degree of difference in gene expression between the two. The larger the |log2FC)|, the more obvious the differential expression of the two genes. The parameter q-value describes the probability that an observed result is a false positive and a smaller q-value mean a more real result. A gene is significantly differentially expressed means |log2FC)|>1 and q-value<0.05. And log2FC>0 means the gene is up-regulated, otherwise means down-regulated.

| **Gene name** | **log2FC** | **q-value** | **Regulation** |
| --- | --- | --- | --- |
| *CLEC17A* | -5.922419081 | 0.024875155 | Down |
| *LOC105378286* | -5.77457481 | 0.033629852 | Down |
| *LINC00977* | -5.521582953 | 0.012625864 | Down |
| *LOC107985728* | -5.145532147 | 0.042388362 | Down |
| *SLC26A11* | -2.076669931 | 0.014205181 | Down |
| *RNF150* | -1.694131713 | 0.008917958 | Down |
| *SIGLEC11* | -1.371737093 | 0.041448021 | Down |
| *ACP3* | -1.275403442 | 0.013937107 | Down |
| *LOC105378665* | -1.234946348 | 0.001446477 | Down |
| *CBFA2T3* | -1.202726459 | 0.02021429 | Down |
| *NUDT7* | -1.172062261 | 0.015311625 | Down |
| *CYSLTR1* | -1.027051669 | 0.001039377 | Down |
| *SLC8A1* | -1.00465749 | 0.015820348 | Down |
| *LOC101928764* | 4.59716049 | 0.033516684 | Up |
| *FOXH1* | 4.375035563 | 0.023026995 | Up |
| *ANGPTL4* | 3.929847331 | 8.61E-09 | Up |
| *HIF1A-AS3* | 3.728356698 | 0.010796457 | Up |
| *TCAF2* | 3.59639377 | 9.16E-17 | Up |
| *BEND5* | 3.289092979 | 0.001007588 | Up |
| *HILPDA-AS1* | 2.850538953 | 6.11E-34 | Up |
| *STC2* | 2.780871246 | 4.38E-12 | Up |
| *CALHM6* | 2.660455721 | 0.003833531 | Up |
| *BCL11B* | 2.499033254 | 0.047209559 | Up |
| *HILPDA* | 2.495702511 | 2.83E-38 | Up |
| *GPR146* | 2.365864091 | 5.54E-05 | Up |
| *MIR210HG* | 2.266174552 | 6.73E-14 | Up |
| *STC1* | 1.84418703 | 0.000191041 | Up |
| *LOC107986977* | 1.809113887 | 0.021476574 | Up |
| *SLC2A1* | 1.787119851 | 1.66E-50 | Up |
| *HK2* | 1.712377055 | 7.47E-41 | Up |
| *NPIPA7* | 1.69473993 | 0.012057716 | Up |
| *ADM* | 1.639611811 | 6.54E-10 | Up |
| *CXCR4* | 1.599071161 | 5.53E-14 | Up |
| *ZNF395* | 1.513334607 | 0.036171447 | Up |
| *TENT5C* | 1.462954379 | 0.000237486 | Up |
| *KDM3A* | 1.267052432 | 3.78E-27 | Up |
| *BNIP3* | 1.262706657 | 4.57E-13 | Up |
| *LOX* | 1.254200969 | 0.00693332 | Up |
| *ANKRD37* | 1.247318114 | 0.00065589 | Up |
| *FLT1* | 1.227389472 | 1.36E-09 | Up |
| *PLAT* | 1.222730105 | 0.003788152 | Up |
| *FUT11* | 1.214135308 | 0.001545408 | Up |
| *PDK1* | 1.204101633 | 2.49E-12 | Up |
| *PLOD2* | 1.167000009 | 0.000143868 | Up |
| *ACHE* | 1.100248907 | 0.028475534 | Up |
| *CCR7* | 1.093253165 | 0.002019025 | Up |
| *HECW2* | 1.055592479 | 0.00741271 | Up |
| *LAMP3* | 1.051817139 | 4.22E-11 | Up |
| *PDGFB* | 1.044054619 | 6.47E-11 | Up |
| *TTYH2* | 1.042460422 | 0.003614427 | Up |
| *MYLIP* | 1.032320834 | 7.48E-05 | Up |
| *ANKZF1* | 1.02315436 | 1.58E-07 | Up |
| *AK4* | 1.010761863 | 7.75E-11 | Up |


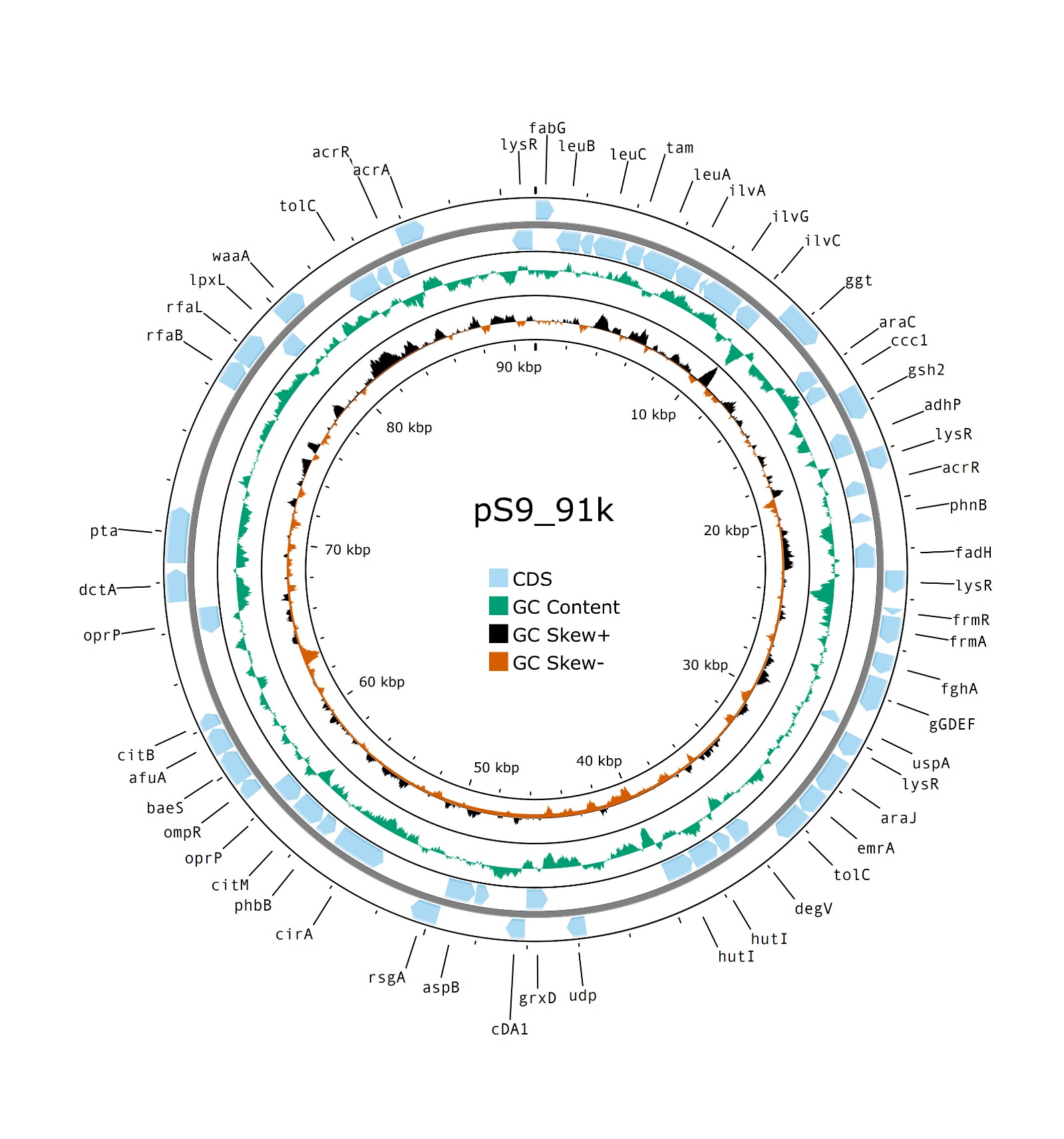


**Figure S1**. **Circos plot of genome of the plasmid in S9.**


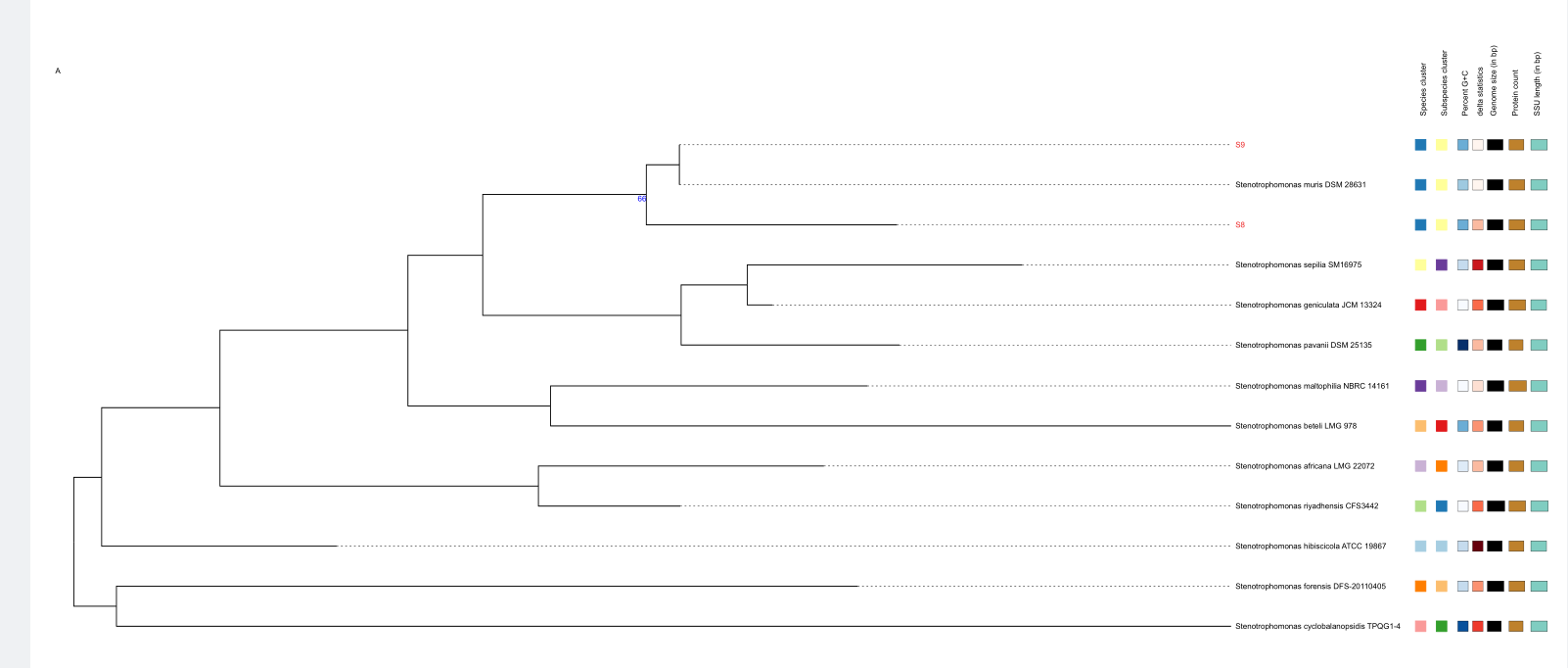

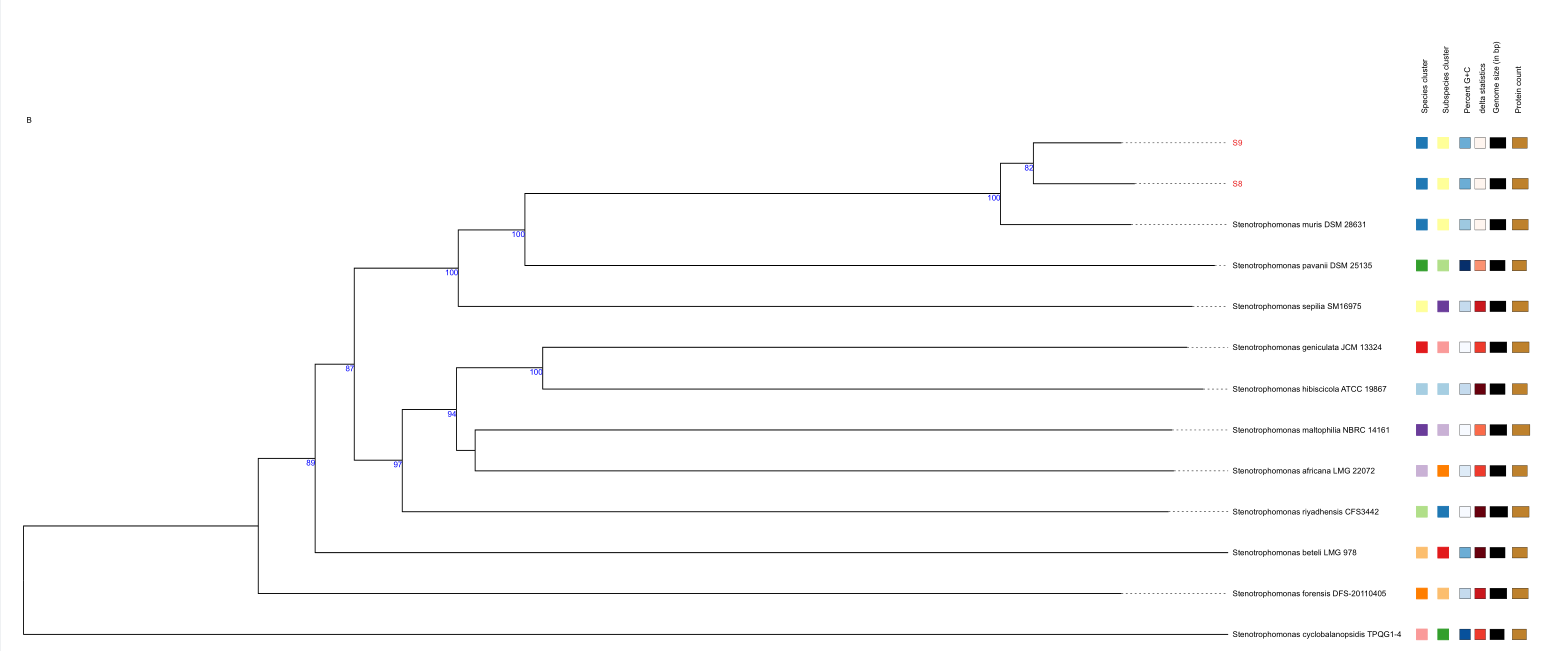


**Figure S2**. **Evolutionary relationships between** **S8/S9 and** ***S. muris*, which act as supplementary evidence that confirms S8 and S9 as *S. muris*.**


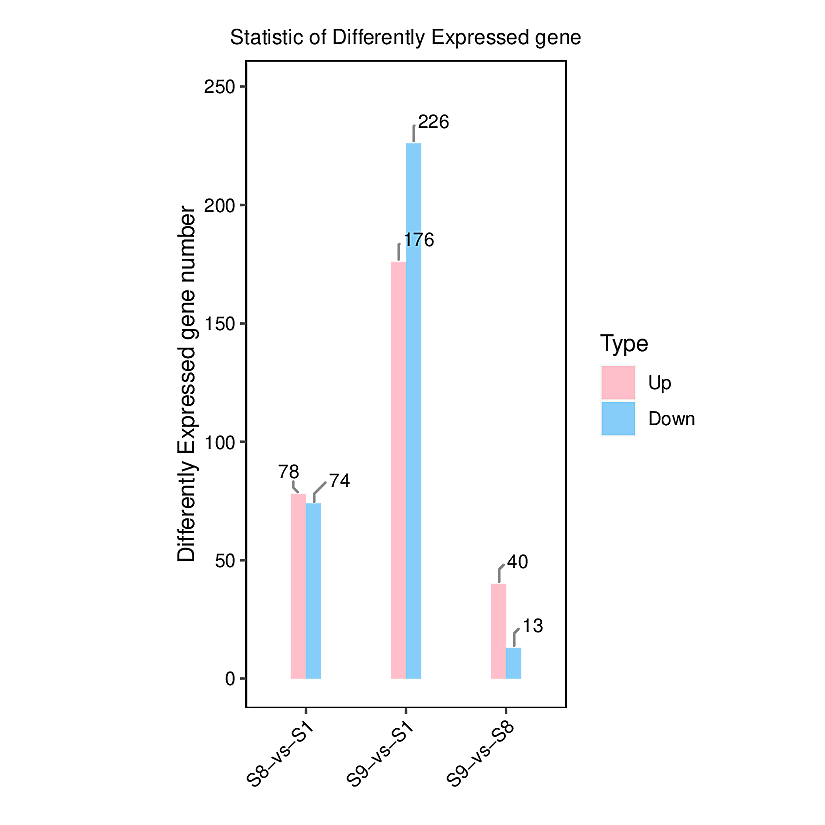


**Figure S3**. **Numbers of differentially expressed genes in S8 versus S1, S9 versus S1 and S9 versus S8 infected cells.**
